# Validation of the japanese version of the sarcoidosis health questionnaire: A cross-sectional study

**DOI:** 10.1186/1477-7525-9-34

**Published:** 2011-05-14

**Authors:** Kiminobu Tanizawa, Tomohiro Handa, Sonoko Nagai, Toru Oga, Takeshi Kubo, Yutaka Ito, Kizuku Watanabe, Kensaku Aihara, Kazuo Chin, Michiaki Mishima, Takateru Izumi

**Affiliations:** 1 Department of Respiratory Medicine, Graduate School of Medicine, Kyoto University, 54 Shogoin Kawaharacho, Sakyo-ku, Kyoto 606-8507, Japan; 2 Department of Rehabilitation Medicine, Kyoto University Hospital, 54 Shogoin Kawaharacho, Sakyo-ku, Kyoto 606-8507, Japan; 3 Kyoto Central Clinic, Clinical Research Center, 56-58 Masuyacho Sanjo-Takakura, Nakagyo-ku, Kyoto 604-8111, Japan; 4 Department of Respiratory Care and Sleep Control Medicine, Graduate School of Medicine, Kyoto University, 54 Shogoin Kawaharacho, Sakyo-ku, Kyoto 606-8507, Japan; 5 Department of Diagnostic Imaging and Nuclear Medicine Graduate School of Medicine, Kyoto University, 54 Shogoin Kawaharacho, Sakyo-ku, Kyoto 606-8507, Japan

## Abstract

**Background:**

Although impaired health-related quality of life (HRQOL) has been reported in patients with sarcoidosis, there is currently no sarcoidosis-specific questionnaire in Japan. The 29-item Sarcoidosis Health Questionnaire (SHQ), originally developed in the United States, is the only sarcoidosis-specific HRQOL questionnaire currently available. The primary aim of this study was to develop and validate a Japanese version of the SHQ.

**Findings:**

The SHQ was translated into Japanese following the forward-backward procedure. The reliability and validity of the Japanese version of the SHQ were examined. One hundred twenty-two Japanese patients with biopsy-proven sarcoidosis were evaluated by the SHQ, the Medical Outcomes Study 36-item short form (SF-36), the St. George's Respiratory Questionnaire (SGRQ), chest radiography, an electrocardiogram, laboratory blood tests, pulmonary function tests, an echocardiogram, and assessments of dyspnea and depressive symptoms. The SHQ was found to have acceptable levels of internal consistency (Cronbach's coefficient α values = 0.68 to 0.91). SHQ scores correlated significantly with scores on the SF-36 and SGRQ. The domain or total scores on the SHQ also significantly correlated with serum levels of the soluble interleukin-2 receptor, the percentage of the predicted forced vital capacity, pulmonary arterial systolic pressure, dyspnea, and depressive symptoms. Also, the SHQ scores of patients who had one or two organ systems affected by sarcoidosis were significantly different from those of patients who had three or more organ systems involvement.

**Conclusions:**

The Japanese version of the SHQ can be used to assess the HRQOL of patients with sarcoidosis.

## Background

Sarcoidosis is a chronic and multisystem granulomatous disease of unknown etiology that can involve almost any organ system [1]. The assessment of overall disease burden is often challenging in sarcoidosis because of the disease's various clinical manifestations. Although spirometry and chest radiography are usually used to evaluate disease activity, these measures fail to address extrapulmonary lesions. As in other respiratory diseases, the patient's health-related quality of life (HRQOL) may be a useful measure to assess the systemic impact of sarcoidosis [2-4], and impaired HRQOL has been reported in sarcoidosis [5]. Given that sarcoidosis is a chronic disease for which there is no curative therapy yet [6], HRQOL could be the most important outcome to measure in the management of sarcoidosis. The incidence of sarcoidosis in Japan is 1.01 per 100,000 inhabitants, and 78.7% of patients are symptomatic at diagnosis [7]. Additionally, sarcoidosis has been listed as one of 130 intractable diseases by the Ministry of Health, Labor and Welfare in Japan. Although more than 1,000 patients are newly diagnosed with sarcoidosis and suffer from the disease, to date, no sarcoidosis-specific HRQOL questionnaire has been developed that can be used for the Japanese population.

The Sarcoidosis Health Questionnaire (SHQ), developed by Cox and coworkers in the United States (US), is the only sarcoidosis-specific HRQOL questionnaire [8]. They reported that the SHQ was more sensitive to differences in the number of involved organ systems than both the generic Medical Outcomes Study 36-item short form (SF-36) [9] and the respiratory-specific St. George's Respiratory Questionnaire (SGRQ) [2]. We therefore hypothesized that the SHQ would also be a useful measurement of the HRQOL of patients with sarcoidosis in Japan. Hence, we aimed to develop and validate a Japanese version of the SHQ.

## Methods

We consecutively enrolled 138 Japanese patients with biopsy-proven sarcoidosis who presented to the Kyoto Central Clinic between June and December 2009. Exclusion criteria included age <18 years, presence of an active neoplasm, and cognitive and/or reading impairment that prevented completion of the questionnaires.

All patients underwent chest radiography, an electrocardiogram, laboratory blood tests, pulmonary function tests (PFTs), and an echocardiogram. The current extent of organ involvement was assessed by the A Case-Control Epidemiologic Study of Sarcoidosis (ACCESS) Organ Involvement Index [10]. Chest radiographs were classified according to standard radiographic staging: stage 0, normal; stage I, bilateral hilar lymphadenopathy (BHL); stage II, BHL with pulmonary infiltrates; stage III, pulmonary infiltrates without BHL; and stage IV, advanced pulmonary fibrosis [11]. Serum angiotensin converting enzyme (sACE) activity and soluble interleukin-2 receptor were measured as they are known to be serum biomarkers of sarcoidosis [12,13]. PFTs were performed by the standardized method [14]. Left ventricular ejection fraction (LVEF) was calculated and pulmonary arterial systolic pressure (PASP) was estimated as described previously [15]. The Institutional Review Board of the Kyoto Central Clinic approved this cross-sectional study, and all patients provided written informed consent.

HRQOL was assessed by the Japanese versions of the SF-36 [9,16] and the SGRQ [2,17]. The Japanese version of the SHQ was developed following the forward-backward translation procedure with independent translations and counter-translations. The SHQ consists of three domains: Daily Functioning (DF), Physical Functioning (PF), and Emotional Functioning (EF). Each item was scored on a 7-point scale, and each domain and the total score were calculated by dividing the sum of the scores by the number of items. Higher scores indicate better HRQOL on the SHQ and SF-36, but worse HRQOL on the SGRQ. Dyspnea was evaluated using the Japanese version of the Medical Research Council (MRC) dyspnea scale in which a higher score indicates a worse status [18]. Depressive symptoms were evaluated by the Japanese version of the Center for Epidemiologic Study-Depression (CES-D, 20-item version) Scale. In this scale, a higher score indicates more depressed symptoms [19,20].

Data are presented as mean ± standard deviation. Cronbach's coefficient α was used to assess the reliability of the questionnaire. Spearman's rank correlation test was used to examine the relationships between SHQ scores and the other HRQOL measures (SF-36 and SGRQ scores) and the clinical variables measured. The Mann-Whitney U test was used to compare HRQOL questionnaire scores between patients who differed in the number of sarcoidosis-affected organ systems (organ system involvement). Values of p < 0.05 were considered statistically significant.

## Results

Of the 138 enrolled patients, 16 were excluded because they either did not complete the questionnaires sufficiently for assessment or they had no active organ involvement of sarcoidosis at that time. Table 1 shows the demographics of the remaining 122 patients and their scores on the SHQ, SF-36, SGRQ, MRC, and CES-D.

**Table 1:** Demographics and HRQOL/clinical findings of the study participants (n = 122)

	Mean ± SD or n (%)	Range
Age, years	56.8 ± 15.5	24 - 85
Female	74 (60.6%)	
Duration of sarcoidosis (months)	108.9 ± 86.2	2 - 395
Number of organ systems involved	1.8 ± 0.8	1 - 5
Radiographic stage	37/32/33/15/5	
(0/I/II/III/IV)	(30.3%/26.2%/27.0%/12.3%/4.1%)	
Blood laboratory tests		
ACE, IU/L	17.2 ± 6.5	3.8 - 41.7
sIL-2R, IU/mL	639.9 ± 507.5	128 - 3772
Pulmonary function tests		
FVC, %predicted	105.8 ± 18.5	44.3 - 146.4
FEV_1_, %predicted	105.8 ± 21.0	35.0 - 158.7
DLCO, %predicted	85.1 ± 17.2	33.7 - 133.3
Echocardiogram		
LVEF, %	68.1 ± 11.4	31.8 - 79.5
PASP, mmHg	29.4 ± 5.9	19.0 - 57.4
SHQ		
Daily Functioning [1-7]	4.7 ± 1.0	2.1 - 6.7
Physical Functioning [1-7]	5.4 ± 1.0	2.7 - 6.8
Emotional Functioning [1-7]	4.8 ± 1.0	2.2 - 6.9
Total [1-7]	4.9 ± 0.9	2.4 - 6.7
SF-36		
Physical functioning [0-100]	79.2 ± 20.9	0 - 100
Role-physical [0-100]	77.1 ± 27.0	0 - 100
Bodily pain [0-100]	71.9 ± 26.7	0 - 100
General health perceptions [0-100]	50.0 ± 19.7	0 - 97
Vitality [0-100]	52.2 ± 23.1	0 - 100
Social functioning [0-100]	78.6 ± 24.1	0 - 100
Role-emotional [0-100]	77.7 ± 25.7	0 - 100
Mental health [0-100]	66.6 ± 20.5	5 - 100
SGRQ		
Symptoms [0-100]	39.8 ± 20.6	0 - 86.5
Activity [0-100]	29.7 ± 24.9	0 - 92.5
Impact [0-100]	14.6 ± 14.6	0 - 57.3
Total [0-100]	23.4 ± 16.3	0 - 71.8
MRC [1-5]	1.8 ± 0.8	1 - 5
CES-D [0-60]	12.8 ± 9.3	0 - 45

The numbers in square brackets represent the theoretical score range.

On the SHQ and SF-36, higher scores indicate better status, while on the SGRQ, MRC, CES-D, lower scores indicate better status.

Abbreviations: HRQOL, health-related quality of life; SD, standard deviation; ACE, angiotensin-converting enzyme; sIL-2R, soluble interleukin-2 receptor; FVC, forced vital capacity; FEV_1_, forced expiratory volume in one second; DLCO, diffusion capacity for carbon monoxide; LVEF, left ventricular ejection fraction; PASP, pulmonary arterial systolic pressure SHQ, Sarcoidosis Health Questionnaire; SF-36, Medical Outcomes Study 36-item short form; SGRQ, St. George's Respiratory Questionnaire; MRC, Medical Research Council; CES-D, Center for Epidemiologic Study-Depression Scale

**Figure 1:**
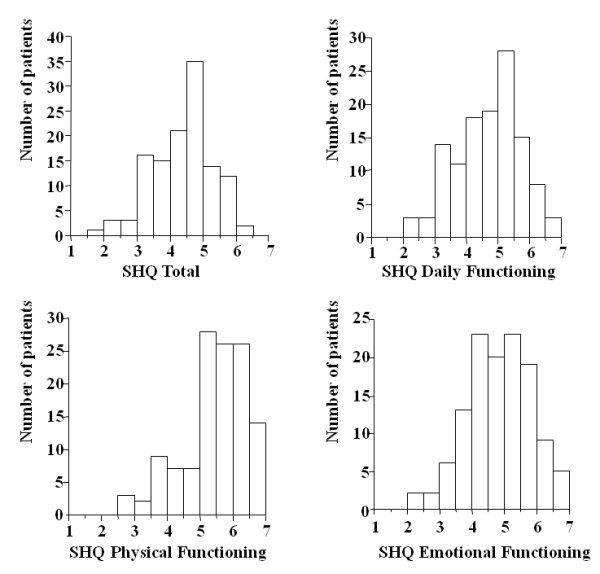
**Frequency distribution histograms of the total and domain scores on the Sarcoidosis Health Questionnaire**. A higher score indicates better health status. The theoretical score range is from 0 to 7 on the total and all domains. Abbreviations: SHQ, Sarcoidosis Health Questionnaire.

Cronbach's coefficient α values of the DF, PF, and EF domains of the SHQ were 0.85, 0.68, and 0.77, respectively, and the α value for the total SHQ score was 0.91. The SHQ total score, as well as the scores for all three domains, were essentially normally distributed (Figure 1).

The relationships between SHQ scores and the other HRQOL measures and the clinical variables measured are presented in Table 2. The three SHQ domain scores and the total score all moderately to strongly correlated with the scores on the SF-36 subscales (Spearman's correlation coefficient, r_s _= 0.45 to 0.78, all p < 0.05). They also weakly to strongly correlated with the three component scores and the total score of the SGRQ (r_s _= -0.74 to -0.27, all p < 0.05). The DF, EF, and total SHQ scores weakly correlated with sIL2-R levels (r_s _= -0.24 to -0.20, all p < 0.05), while the DF, PF, and total SHQ scores correlated with the percentage of the predicted forced vital capacity (%FVC, one of the PFT measurements) (r_s _= 0.18 to 0.27, all p < 0.05), but only the PF score correlated with the PASP (r_s _= -0.38, p < 0.05). All three domain scores and the total SHQ score moderately correlated with scores on the MRC dyspnea scale (r_s _= -0.56 to -0.44, all p < 0.05) and the CES-D scale (r_s _= -0.70 to -0.39, p < 0.05).

**Table 2:** Spearman's rank correlation coefficients between SHQ scores and other HRQOL measures and clinical variables

	SHQ-DF	SHQ-PF	SHQ-EF	SHQ-Total
SF-36				
Physical functioning	0.54	0.66	0.49	0.60
Role-physical	0.70	0.60	0.66	0.74
Bodily pain	0.68	0.68	0.53	0.68
General health perceptions	0.78	0.52	0.66	0.74
Vitality	0.77	0.54	0.70	0.77
Social functioning	0.75	0.49	0.74	0.77
Role-emotional	0.69	0.51	0.69	0.71
Mental health	0.69	0.45	0.71	0.71
SGRQ				
Symptoms	-0.30	-0.48	-0.27	-0.35
Activity	-0.53	-0.71	-0.49	-0.59
Impact	-0.49	-0.60	-0.45	-0.53
Total	-0.55	-0.74	-0.51	-0.61
Radiographic stage	-	-	-	-
ACE	-	-	-	-
sIL2-R	-0.24	-	-0.20	-0.22
%FVC	0.18	0.27	-	0.18
%FEV_1_	-	-	-	-
%DLCO	-	-	-	-
LVEF	-	-	-	-
PASP	-	-0.38	-	-
MRC	-0.44	-0.56	-0.46	-0.50
CES-D	-0.65	-0.39	-0.70	-0.68

Missing data (-) indicate that the relationships were not statistically significant.

Abbreviations: HRQOL, health-related quality of life; DF, Daily Functioning; PF, Physical Functioning; EF, Emotional Functioning; SHQ, Sarcoidosis Health Questionnaire; SF-36, Medical Outcomes Study 36-item short form; SGRQ, St. George's Respiratory Questionnaire; ACE, angiotensin-converting enzyme; sIL-2R, soluble interleukin-2 receptor; FVC, forced vital capacity; FEV_1_, forced expiratory volume in one second; DLCO, diffusion capacity for carbon monoxide; LVEF, left ventricular ejection fraction; PASP, pulmonary arterial systolic pressure; MRC, Medical Research Council; CES-D, Center for Epidemiologic Study-Depression Scale

Patients who had involvement of one or two organ system(s) had significantly lower scores than patients who had three or more organ systems involved in the DF domain (p = 0.01), the EF domain (p = 0.03), the total SHQ score (p = 0.04), and in three of the eight subscales of the SF-36 (p < 0.01 to p = 0.04) (Table 3). The SGRQ scores were not significantly different between the two groups.

**Table 3:** Relationships between different HRQOL measures and the organ system involvement of sarcoidosis

	One or two organs	Three or more organs	p
HRQOL measurements	(n = 102)	(n = 20)	
SHQ			
Daily Functioning	4.8 ± 1.0	4.2 ± 0.9	0.01
Physical Functioning	5.3 ± 0.9	5.3 ± 1.1	0.94
Emotional Functioning	4.9 ± 1.0	4.4 ± 0.7	0.03
Total	4.9 ± 0.9	4.5 ± 0.8	0.04
SF-36			
Physical functioning	81.0 ± 18.5	69.8 ± 29.4	0.08
Role-physical	79.8 ± 25.5	63.4 ± 30.7	0.02
Bodily pain	73.8 ± 25.3	62.0 ± 31.9	0.11
General health perceptions	51.5 ± 20.2	42.3 ± 15.5	0.04
Vitality	53.4 ± 23.1	45.6 ± 22.3	0.18
Social functioning	81.0 ± 21.2	61.9 ± 31.0	<0.01
Role-emotional	79.8 ± 23.3	67.1 ± 34.1	0.17
Mental health	67.9 ± 20.4	59.5 ± 20.2	0.10
SGRQ			
Symptoms	39.6 ± 21.1	41.2 ± 18.7	0.79
Activity	29.1 ± 24.9	32.6 ± 25.1	0.53
Impact	14.2 ± 14.3	17.0 ± 16.3	0.45
Total	22.9 ± 16.3	25.8 ± 16.6	0.44

All data represent mean ± SD.

Abbreviations: HRQOL, health-related quality of life; SHQ, Sarcoidosis Health Questionnaire; SF-36, Medical Outcomes Study 36-item short form; SGRQ, St. George's Respiratory Questionnaire

## Discussion

In this report, we have demonstrated the reliability of a Japanese version of the SHQ and validated it by examining the correlations between SHQ scores and 1) scores on other HRQOL questionnaires and 2) clinical variables in Japanese patients with sarcoidosis.

The reliability of the translated SHQ was assessed for internal consistency. The Cronbach's α values for all three domains and the total SHQ were above the 0.60 required to support construct validity [21], indicating an acceptable level of internal consistency.

The correlations between SHQ scores and SF-36 and SGRQ scores were weakly to strongly significant (|r_s_| = 0.27 to 0.75). These findings indicate that while the translated SHQ evaluated the HRQOL of Japanese patients with sarcoidosis, it did not necessarily measure the same aspects as the other HRQOL questionnaires. The SHQ scores were weakly but significantly associated with a serum biomarker (sIL-2R), pulmonary function, and the echocardiographic index. These correlations indicate that the SHQ can evaluate the effects of multiple pathophysiological processes in sarcoidosis that involve several organ systems. Furthermore, the SHQ scores more highly correlated with patient-reported outcomes such as dyspnea (MRC) and depressive symptoms (CES-D) than with physiological measurements. These findings are consistent with our previous findings in other diseases [17,22,23]. In all, these findings indicate that the SHQ could be an effective assessment for a wide range of the manifestations of the disease, including both functional and psychological.

The validity of the SHQ was also shown by comparing the scores between patients who differed in the extent of organ system involvement. The SHQ scores were significantly different between patients with one or two organ system involvement and those with three or more. Furthermore, compared with the SF-36 and the SGRQ, the SHQ was better able to distinguish the HRQOL between these two groups.

The clinical manifestations of sarcoidosis may differ between different ethnic groups. Compared to Western countries, sarcoidosis in Japan is less severe, but has a higher likelihood of ocular and cardiac involvement [7,24,25]. Thus, HRQOL and its relationship with various clinical features may be different in the different populations. This topic should be explored in the future. Furthermore, one limitation of the study was that the responsiveness of the SHQ over time was not examined, because we did not target those who were newly diagnosed or needed to start treatment as subjects for this study.

## Conclusions

We have demonstrated the reliability and validity of a Japanese version of the SHQ. The SHQ can evaluate the HRQOL of Japanese patients with sarcoidosis, and when added to routine radiological, serological and physiological evaluations of the disease, can provide valuable additional information. As the SHQ is a disease-specific questionnaire, it can evaluate what the generic SF-36 and respiratory-specific SGRQ do not evaluate.

## List of abbreviations

ACCESS: A Case-Control Epidemiologic Study of Sarcoidosis; ACE: angiotensin-coverting enzyme; BHL: bilateral hilar lymphadenopathy; CES-D: Center for Epidemiologic Study-Depression Scale; DF: Daily Functioning; DLCO: diffusion capacity for carbon monoxide; EF: Emotional Functioning; FEV_1_: forced expiratory volume in one second; FVC: forced vital capacity; HRQOL: health-related quality of life; LVEF: left ventricular ejection fraction; SF-36: Medical Outcomes Study 36-item short form; MRC: Medical Research Council; %DLCO: the percentage of the predicted diffusion capacity for carbon monoxide; %FEV_1_: the percentage of the predicted forced expiratory volume in one second; %FVC: the percentage of the predicted forced vital capacity; PF: Physical Functioning; PASP: pulmonary arterial systolic pressure; PFT: pulmonary function tests; sACE: serum angiotensin-coverting enzyme; SHQ: Sarcoidosis Health Questionnaire; sIL-2R: soluble interleukin-2 receptor; SGRQ: St. George's Respiratory Questionnaire.

## Competing interests

The authors declare that they have no competing interests.

## Authors' contributions

Full responsibility for the integrity of the data and the accuracy of the data analysis: TH

Conception and design: TH

Analysis and interpretation of the data: KT, TH, TO

Drafting of the article: KT

Critical revision of the article for important intellectual content: TH, SN, TO, TK,YI, KW, KA, KC, MM, TI

Final approval of the article: SN, KC, MM, TI

Collection and assembly of the data: KT, TH, TK, YI, SN

All authors read and approved the final manuscript.
